# Development and Performance Evaluation of Image-Based Robotic Waxing System for Detailing Automobiles

**DOI:** 10.3390/s18051548

**Published:** 2018-05-14

**Authors:** Chi-Ying Lin, Bing-Cheng Hsu

**Affiliations:** Department of Mechanical Engineering, National Taiwan University of Science and Technology, No. 43, Keelung Rd., Sec. 4, Taipei 106, Taiwan; mstbekzxc@gmail.com

**Keywords:** robotic waxing system, force control, image-based performance evaluation, parameter optimization

## Abstract

Waxing is an important aspect of automobile detailing, aimed at protecting the finish of the car and preventing rust. At present, this delicate work is conducted manually due to the need for iterative adjustments to achieve acceptable quality. This paper presents a robotic waxing system in which surface images are used to evaluate the quality of the finish. An RGB-D camera is used to build a point cloud that details the sheet metal components to enable path planning for a robot manipulator. The robot is equipped with a multi-axis force sensor to measure and control the forces involved in the application and buffing of wax. Images of sheet metal components that were waxed by experienced car detailers were analyzed using image processing algorithms. A Gaussian distribution function and its parameterized values were obtained from the images for use as a performance criterion in evaluating the quality of surfaces prepared by the robotic waxing system. Waxing force and dwell time were optimized using a mathematical model based on the image-based criterion used to measure waxing performance. Experimental results demonstrate the feasibility of the proposed robotic waxing system and image-based performance evaluation scheme.

## 1. Introduction

As a common procedure in auto detailing, waxing removes the fine scratches in automotive sheet metal and reduces surface roughness, which in turn prevents impurities such as dust or moss from sticking to the outer shell of sheet metal, reduces resistance and undesired attachments, and thereby decreases fuel consumption. However, waxing by hand means long durations of applying force, being subjected to machine vibrations, and remaining in poor posture, which cause muscle aches, bone injuries, and damage to the joints in the hand [1]. There are also the issues of uneven force application and inconsistent quality during the waxing process, so waxing quality cannot be guaranteed. This study therefore developed a robotic system for waxing in auto detailing to increase the efficiency and cost-effectiveness of waxing procedures.

At present, most of the existing literature associated with waxing automation lies in patent reports [2,3,4,5], and there are few technical articles that provide data reference. Thus, we based our design on existing robotic systems developed to perform motions similar to those used in waxing, such as grinding [6], deburring [7,8], and polishing [9,10,11,12,13,14]. The objective of those engineering applications was not to alter the geometric shape of the workpiece, but rather to adjust the surface roughness to within set standards. This means that the need for precision in force control far exceeds that required for position control. In previous studies, industrial robotic arms were equipped with force sensors to achieve force control and surface finishing procedures [15,16]. These same devices were also used to measure surface roughness as an indicator of surface quality [10,17]. The researchers in [18] investigated how various parameters pertaining to grinding and polishing processes, including the size of abrasives, the linear velocity of the belt, contact force, and feed rate affected the final surface roughness of the workpiece. Their results revealed that surface roughness is positively correlated with machining force and feed rate. We therefore adopted waxing force and waxing speed (defined as the amount of time spent working on a given spot) as our primary parameters of waxing quality.

One prerequisite for waxing sheet metal is that the surface roughness of the underlying material must surpass a given threshold. Coating quality can be determined using three indices: durability, appearance, and protection against the environment [19]. Protection can be quantified by measuring the contact angle of water drops on the surface of the workpiece [20], wherein a higher angle means that water drops are less likely to adhere to the surface. The measurement of durability involves observing the severity of rust formation following exposure to test environment for extended durations. Appearance (in terms of the numerical classification of paint coatings) can be gauged using tele-spectroradiometers and multi-angle spectrophotometers [21]. Protection and durability depend on the type of coating, but are beyond the scope of this study. We therefore based our assessment of the proposed automated waxing system solely on appearance.

Most auto detailers manually assess and adjust the appearance quality of waxing jobs. Optical microscopes could be used to evaluate the status of workpiece surfaces [10,17,18]; however, this would be time-consuming and expensive. Thus, for the quantification of waxing surface quality, we opted for non-contact image sensing technology, which is easily implemented and inexpensive to install and operate. In [22], image processing algorithms, including thresholding, noise removal, edge detection, and segmentation were used to identify surface defects such as holes, scratches, coil breaks, and rust with a reasonable degree of accuracy. In [23], multilevel thresholds were used to divide grayscale images into various regions representing the roughness of workpiece surfaces as an indicator of surface quality. Although several advanced machine vision based measurement methods have been proposed for assessing surface roughness recently [24,25,26,27], they all suffer from the drawbacks of computational burden or high-cost hardware. In [28], a computer vision system was integrated with a robotic arm to measure surface defects in polished workpieces before and after processing. However, this automated polishing system did not use image feedback to adjust system parameters, such as force or speed to improve processing quality.

In this study, we incorporated computer vision technology within a robotic arm to enable automated waxing while simultaneously monitoring the quality and adjusting system parameters. We developed an image-based assessment method for waxing quality based on image analysis results of wax jobs performed manually by an auto detailer, and used this feedback information to optimize system parameters. Comparing to the existing image-based assessment methods, the proposed method has the advantages of high computational efficiency and easy implementation for the development of autonomous robotic waxing systems. The feasibility of the image assessment criteria and overall performance of the proposed system were evaluated using an engine hood as a waxing target. A depth camera was used to establish point cloud data of the sheet metal in order to plan the waxing path of the system. Waxing procedures were performed using a three-axis robotic arm with an *xyz* platform. Waxing force control was achieved using impedance control and feedback information from a multi-axis force sensor at the end of an effector attached to the robotic arm. The waxing speed was adjusted by altering the dwell time along identical waxing paths. Experimental results confirm the effectiveness of the proposed image assessment criteria in detecting differences in waxing quality. Parameter optimization using this feedback information enabled the proposed waxing system to achieve waxing quality on par with that of professional auto detailers.

## 2. System Description

**Figure 1:**
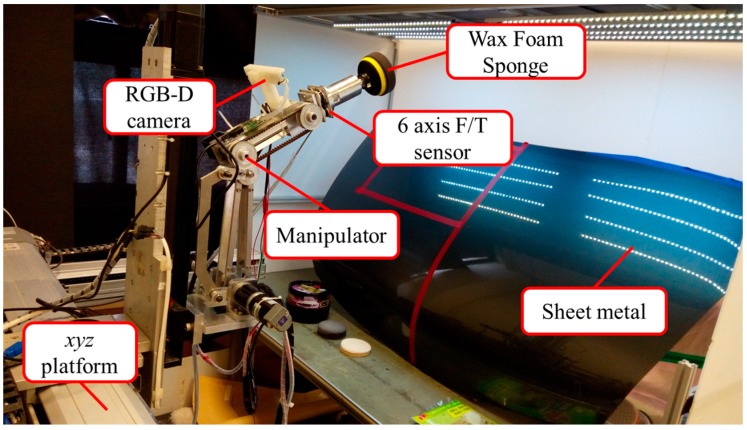
Proposed robotic waxing system.

Figure 1 displays the overall framework of the proposed system. We adopted an *xyz* platform and a self-built three-axis robotic arm equipped with a waxing motor as the end-effector to wax sheet metal. The *xyz* platform is responsible for large-stroke movement. The effective travel range of the platform along the *x*- and *y*-axes is 500 mm × 500 mm, with a screw lead of 10 mm/rev, whereas the effective travel range along the *z*-axis is 400 mm, with a screw lead of 5 mm/rev. The resolution of the motor encoders for each axis is 10,000/rev. The three-axis robotic arm served to wax the sheet metal and exercise force control. DC motors (models IG-42CGM and IG32RGM) manufactured by Sha Yang Ye Industrial Co. Ltd. (Taoyuan, Taiwan) were used for the joints. For the end-effector, we used the DC motor (model GBP30) made by Soho Precision Machinery Co., Ltd. (New Taipei City, Taiwan) to meet the need for greater rotational speed and torque. To use a robotic arm to wax the target sheet metal, we first had to understand the external environment, which includes elements such as posture, position, and color. To do so, we installed an Intel RealSence F200 RGB-D camera (Santa Clara, CA, USA) (color camera with the resolution of 1920 × 1080 pixels; depth camera with the resolution of 640 × 480 pixels) on the second axis of the robotic arm (eye-in-hand setup) to estimate the 3D point cloud and world coordinates of the sheet metal to be waxed and capture post-waxing images to assess waxing quality. We installed a HEX-58-RE-400 N force sensor from OptoForce Ltd. (Budapest, Hungary) on the end-effector to measure waxing force and achieve force control. The system uses a computer to change the position command settings of the *xyz* platform and process the images from the depth camera. The measurement information derived from the force sensor is sent to an Arduino control board via a USB port to control the position of the robotic arm and perform the waxing process.

## 3. Stereo Vision and Path Planning

To obtain the spatial relationship among the target sheet metal, the camera, and the robotic arm and facilitate the waxing process, we first had to establish a coordinate system to calculate the correct location of the sheet metal and plan the waxing path [29]. We employed a depth camera to obtain the color and depth information of the sheet metal. The parts that do not belong to the sheet metal are removed via image preprocessing, and then the infrared reflection principle of depth cameras is utilized to achieve stereo vision, estimate the point cloud plot of the target, and plan the system’s waxing path.

### 3.1. Image Processing

**Figure 2:**
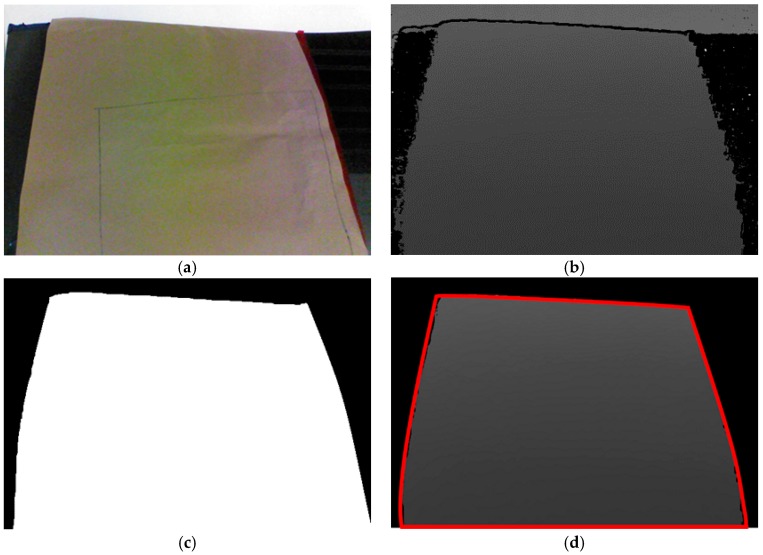
Mask operation. (**a**) color image; (**b**) depth image; (**c**) mask computing; (**d**) mask to depth image.

Estimation of the sheet metal’s location is based on color images from the depth camera, which are integrated with the information measured from the camera plane to establish the point cloud plot of the sheet metal. However, each point cloud plot contains three channels of image information and depth information for all the coordinates in the images, so the information content is substantial. To reduce the computation time, the color images from the depth camera are subjected to color filtering, thresholding, and morphology processing. The resulting black-and-white images serve as masks to remove the areas that do not belong to the sheet metal in the images, which reduces the system’s computation time and decreases the chance of miscalculations. The mask operation procedure is as shown in Figure 2. Before capturing images, we covered the sheet metal with non-reflective khaki paper to prevent incomplete depth information extraction due to reflections, as shown in Figure 2a. Figure 2b displays the depth image information that was captured. The shade of the color indicates the distance of a spot from the camera. The scattered back spots were due to the diffusion of infrared light caused by the sheet metal, so the camera could not receive the light reflected back from these posts, which resulted in incorrect depth values. Figure 2c presents the mask created via image processing. The white area is the part that the system needs to wax and the target of coordinate conversion, whereas the black area does not need to be processed. After placing this mask on the depth plane, what remains is the part needed by the system, as shown in Figure 2d. Converting the coordinates of the depth information and location of each spot then produces the point cloud plot of the sheet metal.

### 3.2. Stereo Vision

**Figure 3:**
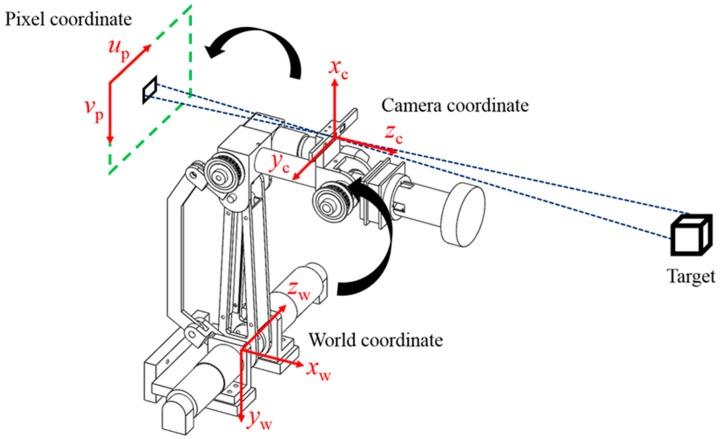
Coordinate conversion.

The locations of the robotic arm, camera, and target are not fixed, so the relationships of the three with the world coordinates have to be established [30]. We installed a depth camera on the second axis of the robotic arm to create a stereo vision system. Based on different descriptive backgrounds, the system has to define three different coordinate systems to describe the relationships among the target, the camera, and the robotic arm. These coordinate systems include the world coordinate system (*x_w_*, *y_w_*, *z_w_*), the camera coordinate system (*x_c_*, *y_c_*, *z_c_*), and the pixel coordinate system (*u_p_*, *v_p_*). The conversion relationship is as shown in Figure 3. The world coordinate system refers to a specific origin in the workspace, so any object in the space has coordinates in relation to the defined origin so that the world coordinate system and robotic arm coordinate system can be defined with an identical origin and directions. The camera coordinate system is a coordinate system that describes other objects based on the location of the camera. Generally speaking, there is no deflection in the light that travels through the center of the lenses. For this reason, we set the center of the lenses as the origin of the camera coordinate system. The origin of the pixel coordinate system is the upper left corner of the image plane and describes the location of the target on the image plane. The system must convert the location of the target into world coordinates so that the robotic arm can track the planned path. The coordinate conversion is achieved by first converting the world coordinates into camera coordinates and then converting them into pixel coordinates.

The conversion relationship between the pixel coordinates and the world coordinates is as presented in Equation (1):

(1) $$S\left[\begin{array}{c} u_{p}\\ v_{p}\\ 1 \end{array}\right]=\left[\begin{array}{cccc} f & 0 & 0 & u_{i}\\ 0 & f & 0 & v_{i}\\ 0 & 0 & 1 & 0 \end{array}\right]\left[\begin{array}{cc} R_{3\times 3} & T_{3\times 1}\\ 0_{1\times 3} & 1 \end{array}\right]\left[\begin{array}{c} x_{w}\\ y_{w}\\ z_{w}\\ 1 \end{array}\right],$$

where *S* is a magnification factor; (*u_p_*, *v_p_*) are the target coordinates as described by the pixel coordinate system, and (*x_w_*, *y_w_*, *z_w_*) are the coordinates of the target in the world coordinate system; *f*, *u_i_*, and *v_i_* are intrinsic parameters of the camera, which are fixed upon manufacturing; *f* denotes the focus of the camera; (*u_i_*, *v_i_*) are the coordinates of the camera center. Note that these intrinsic parameter values can be obtained via a standard camera calibration process [31]. **R**_3×3_ and **T**_3×1_ are the extrinsic parameters of the camera, including a translation matrix and a rotation matrix. However, extrinsic parameters vary with world coordinate definitions and the position in which the camera is installed. We installed the camera on the second axis of the robotic arm in this study, so the conversion relationships between coordinate systems will change as the robotic arm moves. Thus, a camera coordinate system had to be defined to calculate the extrinsic parameters.

**Figure 4:**
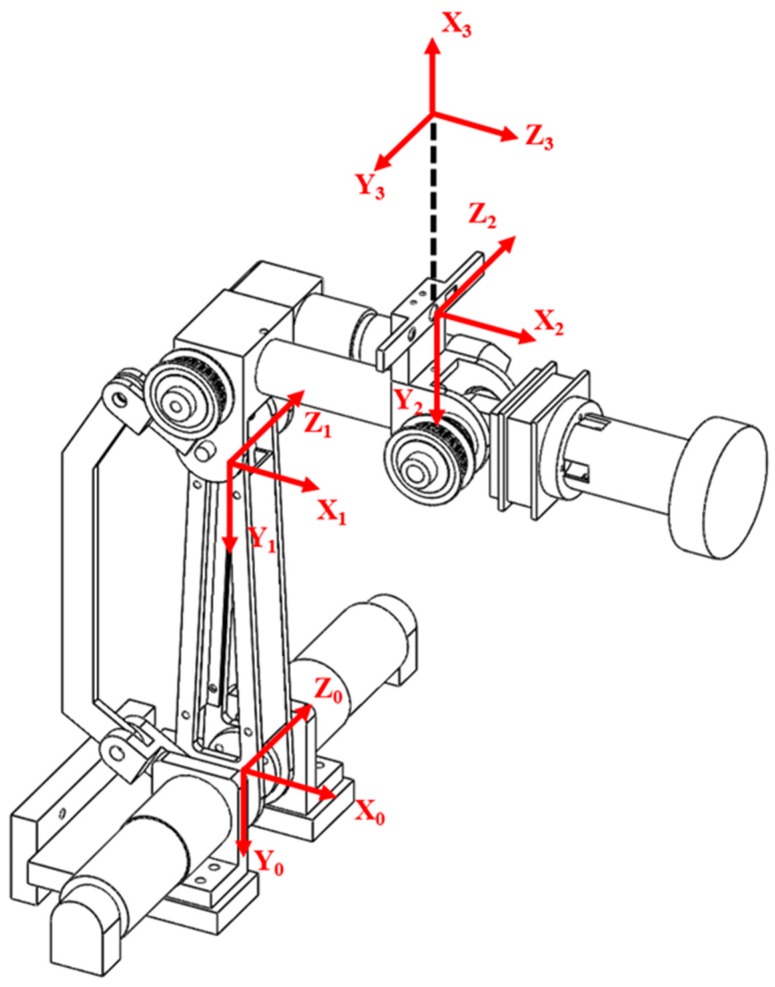
Camera coordinate system (Frame 0–1: motor rotation axis, Frame 2: position of camera, Frame 3: center of camera).

The axes of the robotic arm and the camera coordinate system are as shown in Figure 4. Frames 0 and 1 are the coordinate systems of the first and second axes of the robotic arm and change with motor rotations. Frames 2 and 3, respectively, represent the position of the camera and the center coordinates of the camera and do not change with the posture of the robotic arm. According to this coordinate system, we can derive the conversion relationship in Equation (2), where **E** is the extrinsic parameter of the camera; **P_r_** and **P_c_** are the locations of the target as described by the coordinate systems of the robotic arm and the camera, respectively. With this coordination matrix, we can convert the target location estimated by the camera coordinate system into coordinates in the robotic arm coordinate system, perform 3D reconstruction using the depth and color planes obtained using the depth camera, and construct a point cloud plot of the target sheet metal to plan the waxing path:

(2) $$P_{r}=EP_{c}.$$

### 3.3. Planning of Waxing Path

**Figure 5:**
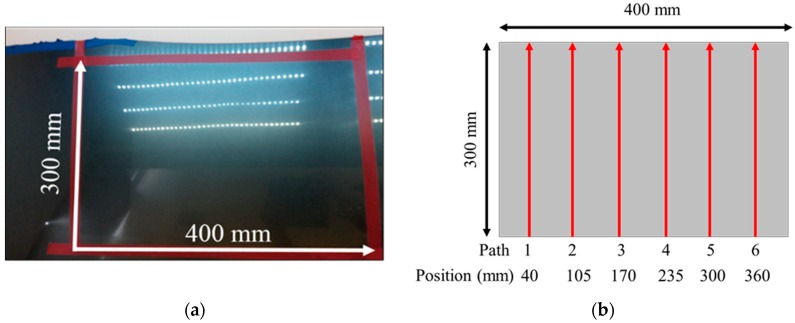
Path planning for the sheet metal. (**a**) area to be waxed; (**b**) waxing trajectories.

**Figure 6:**
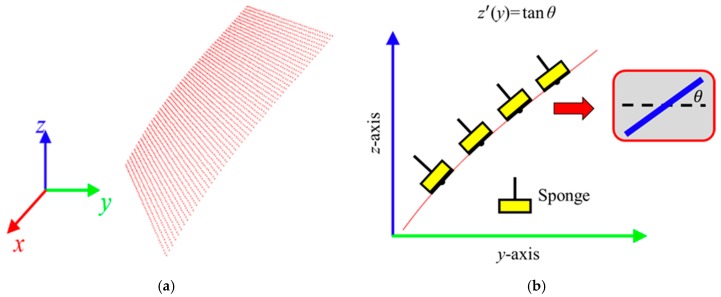
Posture constraint for the robot end-effector. (**a**) point cloud 3D plot; (**b**) slope estimation.

The planar dimensions of the target plane on the engine hood to be waxed in this study was 400 mm × 300 mm, as shown in Figure 5. During the waxing procedure, each swipe overlapped the previous swipe to a certain degree so as to prevent missing any spots. In order to evenly cover each spot on the target area, the system divided the waxing path into six paths, and waxing was performed from the bottom upwards. The relative positions of each path were at 40 mm, 105 mm, 170 mm, 235 mm, 300 mm, and 360 mm, as shown in Figure 5, and waxing was performed with a waxing sponge 80 mm in diameter, so the paths overlapped about 1/5 of each other. We conducted curve fitting of the different paths three times based on the side view of the *yz* plane of the point cloud plot converted from the coordinates (as shown in Figure 6). The results are as shown in Table 1. We then calculated the derivative of the curve equation:

(3) z′(y)=tanθ,

with the equation above, we could calculate the angle *θ* between the horizontal plane and the tangents at different spots on the paths on the surface. With inverse kinematics, we established the posture limitations of the robotic arm to ensure that the wax can coat the sheet metal evenly without angle deviations that prevent the robotic arm from applying force vertically to the curved surface of the sheet metal.

## 4. Force Control of Robotic Arm

**Figure 7:**
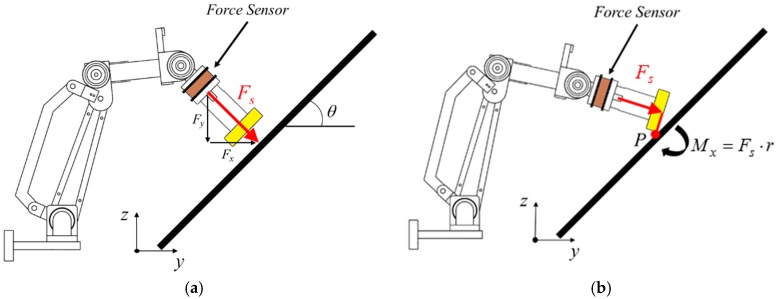
Use of robotic arm in waxing force control. (**a**) normal waxing force control; (**b**) end-effector not flat on sheet metal.

**Figure 8:**
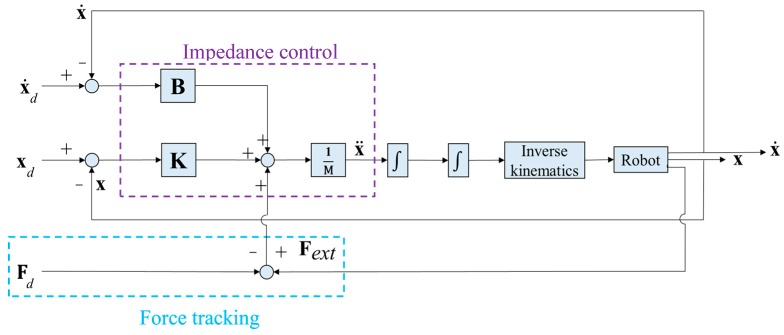
Block diagram of robot force control system in this study.

For the robotic arm to maintain constant force while waxing and avoid damaging the sheet metal and the structure of the robotic arm, we adopted an impedance control method that is commonly seen in the literature to control the waxing force of the robotic arm [32]. The concept is to achieve force tracking control with the expected force of the system (**F***_d_*) and an impedance control algorithm. Furthermore, if the sponge at the end-effector of the robotic arm is not completely flat on the surface of the sheet metal, it will affect the evenness of force application, increase the chance of structural damage in the robotic arm, and create an additional moment *M_x_* in the robotic arm, as shown in Figure 7. In view of this, we also considered orientation impedance control to keep the sponge flat on the surface based on the external moment. Figure 8 displays a block diagram of the overall force control system. Considering the position terms *p_y_* and *p_z_* of the end-effector in the *y*- and *z*-directions and rotation angle *θ_x_* around the *x*-axis, **x** = [*p_y_ p_z_ θ_x_*]*^T^* (position of end-effector); $\dot{x}$ and $\ddot{x}$ denote the velocity and acceleration of the end-effector, **x***_d_*= [*p_yd_ p_zd_ θ_xd_*]*^T^* is the desired position of the end-effector. The mass and moment of inertia matrix of the end-effector is **M** = *diag*(*M_y_*, *M_z_*, *J_θ_*), and the matrices of damping and stiffness between the robotic arm and the environment are **B** = *diag*(*B_y_*, *B_z_*, *B_θ_*) and **K** = *diag*(*K_y_*, *K_z_*, *K_θ_*), respectively. The force that comes into contact with the external environment is the external force/torque **F***_ext_*= [*F_y_ F_z_ M_x_*]*^T^*. As the force sensor is installed on the end-effector, conversion relationships *F_y_* = *F_s_*sin*θ* and *F_z_* = −*F_s_*cos*θ* exist between the measured waxing force *F_s_* and the coordinate system of the point cloud. Note that *θ* can be derived using Equation (3). Let the waxing force being applied to the target be *F_w_*; its components *F_yd_* = *F_w_*sin*θ*, *F_zd_* = −*F_w_*cos*θ*, and *M_xd_* = 0 can be expressed using expected force vector **F***_d_*= [*F_yd_ F_zd_ M_xd_*]*^T^*.

The principle behind Figure 8 is to use the difference between **F***_ext_* and **F***_d_* to change the position responses of the overall system. When the difference is zero, it means that the force applied by the robotic arm to the sheet metal has reached the system's target value. Furthermore, impedance control is used to alleviate the impact of the force created by the end-effector when it comes into contact with the sheet metal.

## 5. Image Based Performance Evaluation

**Figure 9:**
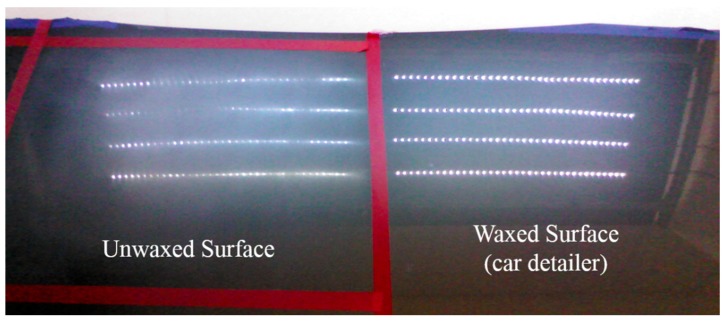
Comparison of LED lighting results.

To effectively test the waxing results and verify the feasibility of the proposed system, we divided the automotive sheet metal used in our experiment into two parts as shown in Figure 9. The left part was waxed by the robotic arm, while the right part was waxed by an experienced auto detailer, which served as the control group. Using the auto detailer’s waxing results, we applied an external light source for analysis to develop assessment criteria for waxing quality.

### 5.1. Selection of External Light Source

White point light sources have a condensation effect that shows any fine scratches on paint surfaces. For this reason, we adopted eight 5050 LED strips 50 cm in length for the light source. Each strip contained 36 LED point lights, so there were 144 point lights on either side, as shown in Figure 9. The right shows the reflection results of the waxing done by the auto detailer. As the fine scratches on the surface of the sheet metal have been filled in, the result is an almost mirror-like reflection of the white point light sources, and each point light can be distinguished clearly. In contrast, the fine scratches in the unwaxed area on the left are still very visible, so the reflection results are dim, and some reflected light spots are indistinguishable with only a glimmer remaining.

### 5.2. Waxing Assessment Criteria

To evaluate the quality of waxing in the waxed areas, we utilized the condensation effects of the round LED point lights. Image preprocessing was conducted on the area waxed by the auto detailer to calculate the radius distribution of the reflected light spots. The preprocessing included grayscale conversion, Canny edge detection [33], and the Hough circle transform [34]. Next, we calculated the radius of each reflected light spot for statistical analysis to establish the assessment criteria of the proposed waxing system.

#### 5.2.1. Image Preprocessing

**Figure 10:**
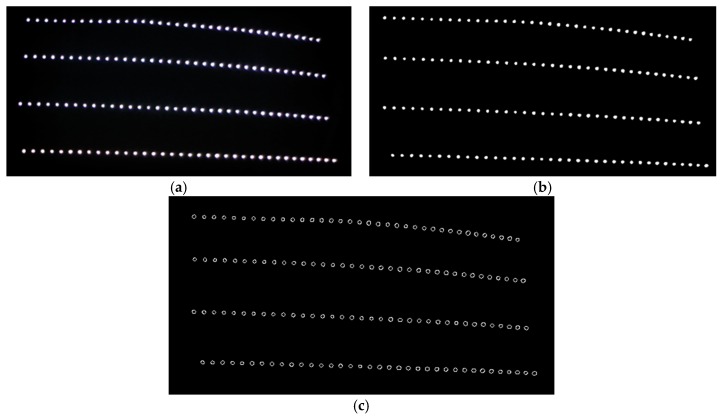
Results of image preprocessing (area waxed by auto detailer). (**a**) captured RGB image; (**b**) RGB to grey image; (**c**) after edge detection.

Once the camera captures a color image of the waxed surface, it must be converted to grayscale for edge detection. Grayscale conversion merges the three-color channels into a single channel, and it is also a way of expressing the brightness values of each pixel. The image processing results are as shown in Figure 10. Figure 10a shows the image captured by the camera of the surface waxed by the auto detailer; Figure 10b displays the image following grayscale conversion; Figure 10c presents the results of edge detection. As can be seen, the halos in the image were completely removed after edge detection. Next, the Hough circle transform determines whether the reflected light spots are uniform in size and presents their distribution conditions.

**Figure 11:**
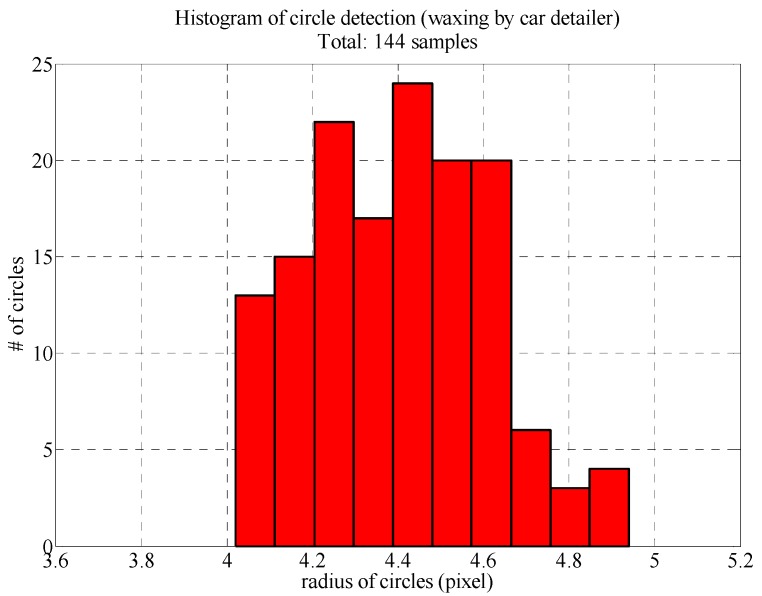
Histogram of reflected LED light spot radiuses (area waxed by auto detailer).

Figure 11 presents the radius distributions of the reflected LED light spots following the Hough circle transform. All 144 reflected light spots were circular and 4 pixels to 5 pixels in diameter. The maximum error was less than 1 pixel, which means that the auto detailer had filled in the fine scratches on the surface of the sheet metal, so the resulting surface facilitated reflection. The influence of the poor surface conditions on the image analysis results was also reduced.

#### 5.2.2. Establishment of Image Assessment Criteria

To effectively analyze the radius distributions resulting from circle detection, we used the mean and standard deviation to construct the probability density function of the normal distribution and express the degree of dispersion in the radiuses of the reflected LED light spots. The distribution conditions of this function served as the assessment criteria. If the image analysis results of the area waxed by the proposed robotic arm meet the preset threshold, it means that the proposed system has successfully completed the waxing process.

**Figure 12:**
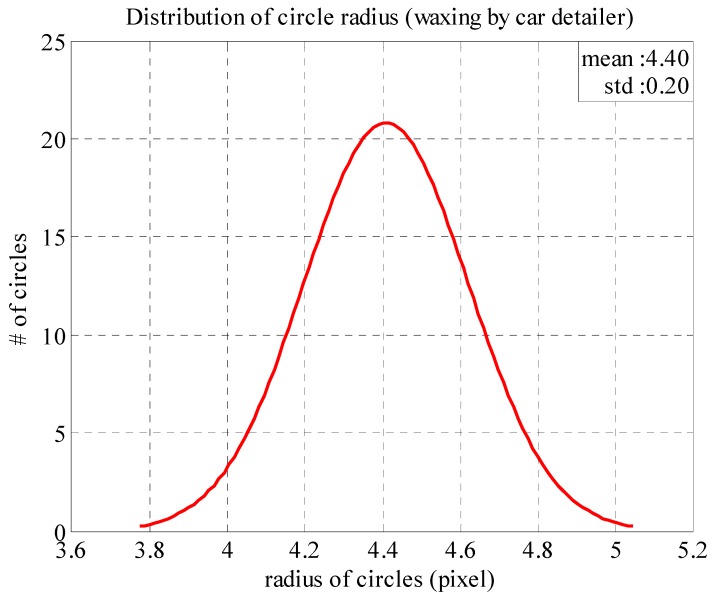
Normal distribution of reflected light spot radiuses (area waxed by auto detailer).

Figure 12 presents the normal distribution function plotted based on the radius distribution from the area waxed by the auto detailer. The mean ($\bar{x}$) and standard deviation (*σ*) of the function are 4.40 and 0.20, respectively. As the standard deviation is very small, it is clear that the function distribution is very close to the mean of the series. This means that the area waxed by the auto detailer was very smooth and could completely reflect the LED light source above to the camera plane; no fine scratches caused light diffusion.

**Figure 13:**
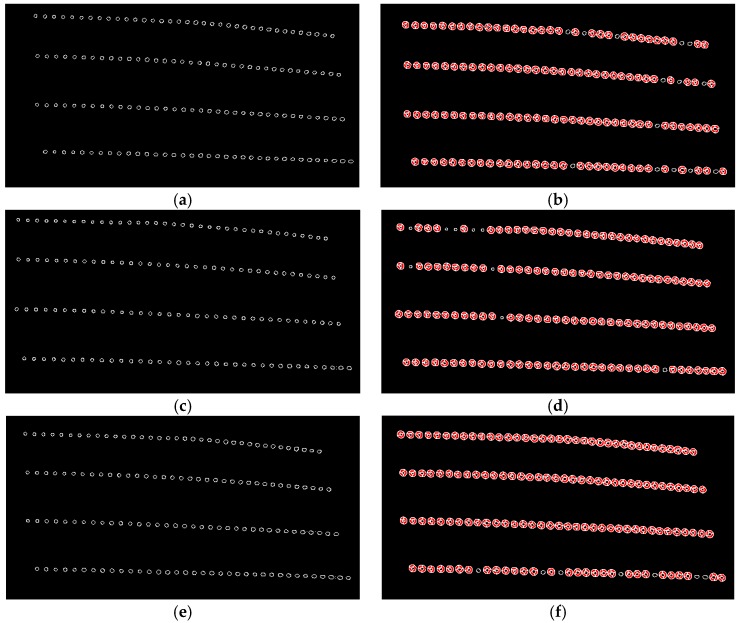
Image analysis results from different camera angles. (**a**) circle detection results from camera angle 15°; (**b**) circle screening results from camera angle 15° (130 samples); (**c**) circle detection results from camera angle 30°; (**d**) circle screening results from camera angle 30° (135 samples); (**e**) circle detection results from camera angle 45°; (**f**) circle screening results from camera angle 45° (137 samples).

The data above was obtained from a camera angle of 30°.To confirm whether these statistical characteristics can serve as the assessment criteria of the system, we analyzed the area waxed by the auto detailer using images taken from camera angles of 15°, 30° and 45° and used the data from the 30° images for quality screening. In the following experiments, we used an intermediate value of camera angle (30°) to ensure desired reflection effects and avoid unexpected overexposure for consistent image analysis results. Table 2 displays the radius analysis results from the different angles, and Figure 13 shows the corresponding image analysis results. The red circles indicate that the circle meets the threshold standard. As can be seen, the results from the different angles are very similar, which demonstrates that the data can serve as the assessment criteria for waxing quality. We set the mean plus and minus two standard deviations (±2*σ*) as the acceptable range for reflected light spot radiuses. Waxing results that are closer to the average value of the ones in Table 2 (134 samples) indicate greater quality, which is the objective of the proposed system.

## 6. Experiments and Performance Analysis

The experiment in this study was divided into two parts: force tracking and wax image analysis. Due to the fact that force tracking performance will affect the evenness of the wax covering the sheet metal while the robotic arm is waxing, the impedance control parameters must be adjusted in advance so that the waxing force of the robotic arm will remain constant and thereby prevent excessive error from affecting waxing quality or damaging the robotic arm or target sheet metal. Once suitable impedance parameters are obtained, a waxing experiment is conducted using these parameters with waxing force and dwell time as the impact factors of waxing quality so as to analyze and investigate the waxing results derived from different parameters and the pre-established assessment criteria.

### 6.1. Analysis of Force Tracking Performance

The impedance control parameters (**M**, **B** and **K**) in Figure 8 all influence the force tracking error and response speed. By adjusting the impedance parameters, we change the response behavior of the system to achieve force tracking performance with stability, a short settling time, and no overshoot. If the damping ratio (*ζ*) is set as 1 and the settling time as less than 0.5 s, then, with the transient responses of a standard second-order dynamic system [35], we can derive that the natural frequency (*ω_n_*) of the system must be greater than 8 rad/s to fulfill this constraint. In this experiment, we set the natural frequency as 10 rad/s; in terms of *F_y_*, if the mass coefficient selected for the impedance controller is *M* = 3.5 kg, then the damping and stiffness coefficients are *B* = 70 N s/m and *K* = 350 N/m, respectively. We also chose the same set of parameters to achieve the impedance control of components *F_y_* and *M_x_* and used a single target point to perform waxing force tracking of the robotic arm and analyze its response results.

**Figure 14:**
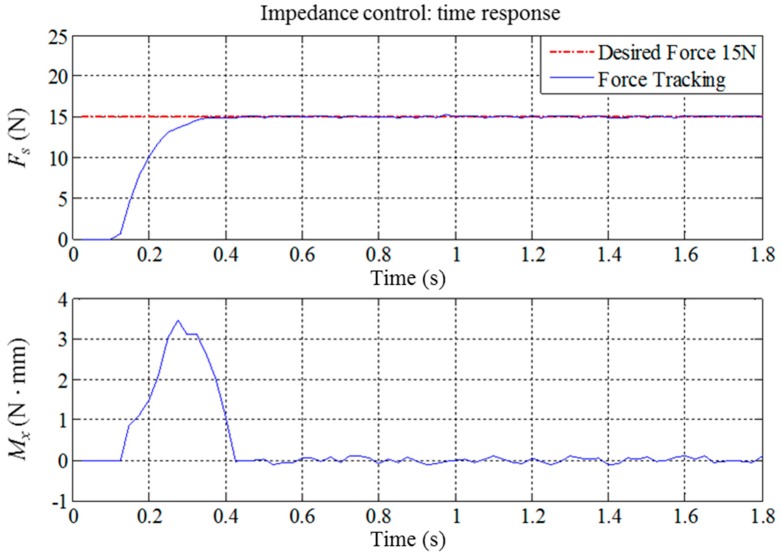
Force tracking control results of the robotic waxing system (*F_w_* = 15, **M**_3×3_ = *diag*(3.5, 3.5, 3.5), **B**_3×3_ = *diag*(70, 70, 70), **K**_3×3_ = *diag*(350, 350, 350)).

As previously shown in Figure 7, when the system begins the waxing process, the end-effector must maintain vertical contact with the surface of the sheet metal to ensure that the waxing sponge is completely flat on the sheet metal. Thus, we must consider whether *F_s_*, the force that is vertical to the sheet metal, reaches the target value *F_w_* set by the system. At the same time, we must suppress the torque *M_x_* created when the end-effector is not vertical to the sheet metal. Figure 14 presents the response results of force tracking when *F_w_* is set as 15 N. As can be seen, the responses resulting from this set of parameters can quickly reach the target value, the settling time is significantly shorter than 0.5 s, no overshoot exists, and the absolute value of the steady state error is less than 0.3 N. The robotic arm showed some posture deviations during the time from 0.1 s to 0.4 s, which prevented the end-effector from being completely flat on the sheet metal surface and created a torque of approximately 3 N mm. However, posture compensation eliminated this after 0.4 s, so it did not affect the results of the waxing experiment. When *F_w_* is set between 10 N and 20 N, the force tracking results of other fixed values present an identical trend.

### 6.2. Examination of Waxing Parameters

In this experiment, we divided the area to be waxed on the sheet metal into six paths. Based on the previously established waxing paths, we adjusted the dwell time of each spot to change the waxing speed. We also used the impedance parameters established in Section 6.1 for waxing force control. After the waxing task was completed, we waited for 5 to 10 min for the wax to dry and solidify. To simplify the overall experimental process, a normal cotton buffing cloth was used to remove the excess wax (the dewaxing/polishing process). With the camera angle set at 30°, images of the reflections of external point lights resulting from different experimental conditions were processed, and the radius of each reflected light spot was calculated. The results were then compared with the assessment criteria that we established to verify the feasibility of the proposed system. The accepted range of circle radiuses and the target number are indicated in Table 3, the latter being 134 circles and the threshold set at a difference under 10 circles.

#### 6.2.1. Experiment Conditions and Parameter Settings

We used Carnauba wax, which is the most common type of wax used in the auto detailing industry, for the waxing experiment. The waxing sponge, a fine waxing sponge specifically made for auto detailing, was 25 mm thick to prevent the vibrations caused by waxing from damaging the structure of the robotic arm, facilitate heat dissipation needed for high-speed motor rotations, and reduce the possibility of burning the wax and the sheet metal surface. Table 4 presents the waxing parameter settings of the system. As the force required in most waxing procedures ranges from 10 N to 20 N, we experimented with waxing forces 10 N, 13 N, 15 N, 17 N, and 19 N and chose a motor with rotational speed 850 rpm and torque output reaching 30 N-m for the waxing motor. For dwell time, we experimented with 0.1 s/pt, 0.2 s/pt, 0.3 s/pt, and 0.4 s/pt to facilitate subsequent investigations. In the experiment, we divided the *y*-direction into 500 equally spaced points (as shown in Figure 6) and used the curve fitting equation and Equation (3) in Section 3.3 to derive the target location of the end-effector, ***x_d_***. Via inverse kinematics, we obtained the motor position command and then realized point-to-point motion control in the motor with the proportional-derivative (PD) control algorithm. Waxing of each fixed point was then performed from the bottom upwards according to each of the planned paths. The movements between paths were achieved using the *xyz* platform, coordinated with impedance control to prevent the robotic arm from applying excessive force when it comes into contact with the sheet metal.

#### 6.2.2. Discussion of Waxing Results

**Figure 15:**
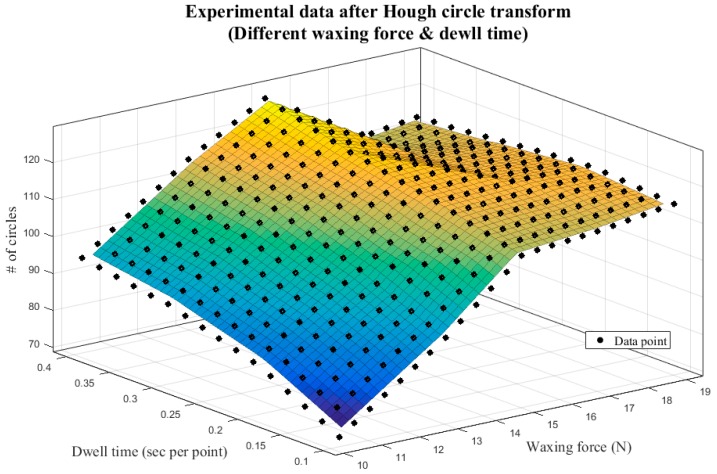
Experimental results of different waxing parameters—3D plot.

Table 5 presents the image analysis results from the five different waxing forces above, and Figure 15 displays the corresponding 3D plot. Observation revealed that, as the waxing force increased from 10 N to 15 N, the number of reflected light spots increased significantly because the wax filled the fine scratches more fully as the waxing force increased. Once the waxing force reached 17 N; however, the number of assessment criteria that met standards stagnated and began to decline. This is because the optimal waxing force for the Carnauba wax chosen for this system is around 15 N; excessive force affects the stacking effect of the wax on the sheet metal, preventing the wax from filling in the fine scratches normally and leading to the diffusion of the reflected light spots on the sheet metal surface. As a result, the image analysis results were poorer than those at 15 N.

As can be seen in Figure 15, the number of circles that meet standards increases with dwell time and peaks at 127 when waxing force 15 N is paired with 0.4 s/pt. This number is less than the result shown in Table 3 by seven circles and within the threshold of 10 circles, which means that the quality is already very close to that achieved by the auto detailer. When the waxing force is greater than 15 N, the image analysis results do not improve as the dwell time increases. The reason is similar; excessive force affects the stacking effect of the wax on the sheet metal, and even if the waxing speed decreases, the wax cannot fill in the scratches on the sheet metal, which reduces the repairing effects of the wax. Thus, the results of increased dwell time paired with waxing forces 17 N and 19 N were poorer than those with waxing force 15 N.

### 6.3. Waxing Parameter Optimization

The surface formed by the results in Figure 15 presents a convex function. To derive the optimal parameters, we conducted 3D surface fitting with the experimental results from different waxing forces using least squares regression, thereby producing a mathematical model for optimization.

#### 6.3.1. Problem Formulation

**Figure 16:**
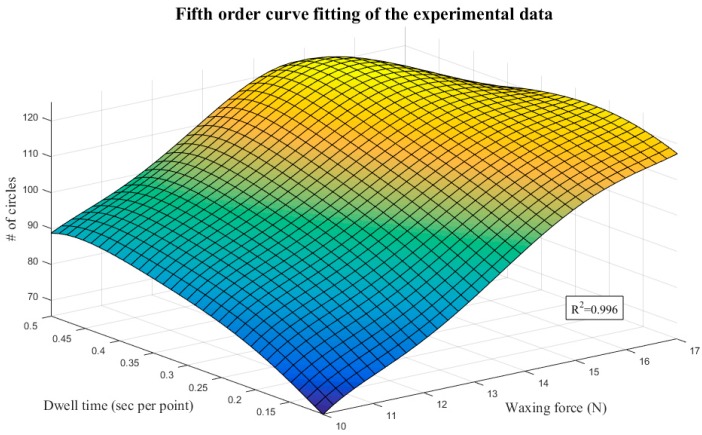
Fifth-order surface fitting results.

The mathematical model for optimization comprises two parts: an objective function and constraints. Both parts are functions of the design variables. We adopted a fifth-order polynomial to fit the surface using least squares regression, as shown in Equation (4). Figure 16 presents the surface fitting results of the experimental data. The R-squared value (R^2^ = 0.996) indicates that the fitted surface is very close to the distribution of the experimental results, so the quantic equation with two variables served as the objective function of optimization in the proposed system. The various coefficients are as shown in Table 6:

(4) $$z=f(x,y)=\sum_{n=0}^{5}\sum_{i=0}^{n}p_{\left(n-i,i\right)}x^{n-i}y^{i}.$$

Table 7 presents the parameter constraints of the optimization problem. As mentioned in the previous section, excessive force is not suitable for the car wax chosen for this system. For this reason, we limited the waxing force to between 10 N and 17 N. In consideration of the overall efficiency of the system, we limited the dwell time to between 0.1 s/pt and 0.5 s/pt so as to prevent overly long dwell time from burning or wearing down the wax, which would then prevent the image analysis results from corresponding to the optimized parameters.

#### 6.3.2. Optimization Results and Verification

We chose constrained nonlinear minimization [36] for parameter optimization and used the Matlab Optimization Toolbox (version 8.0, The MathWorks, Inc., Natick, MA, USA) to derive the optimal combination of waxing parameters. Constrained nonlinear minimization searches for the minimum value of the objective function within the constraints. Thus, we multiplied Equation (4) by −1 to flip the surface, as shown in Equation (5), to facilitate optimization:

(5) $$\mathrm{Objective}\;\mathrm{function}:\;z=-f(x,y)=-\sum_{n=0}^{5}\sum_{i=0}^{n}p_{\left(n-i,i\right)}x^{n-i}y^{i}.$$

The optimization process above included six iterations and 26 calculations. The results indicate that waxing force 15 N and dwell time 0.5 s/pt is the optimal parameter combination of the system. The number of reflected light spot radiuses that reached the assessment criterion was 132. Next, we performed the waxing experiment using this parameter combination, conducted image analysis of the results, and compared them with the assessment criteria.

**Figure 17:**
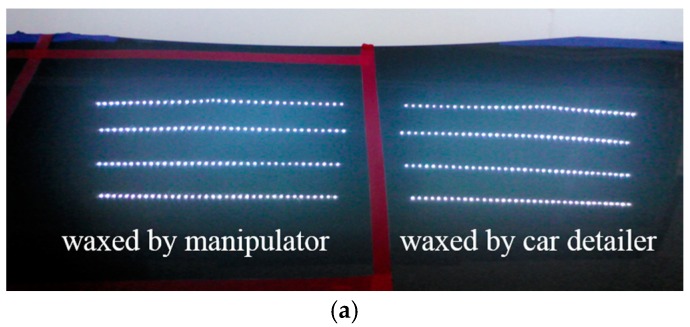

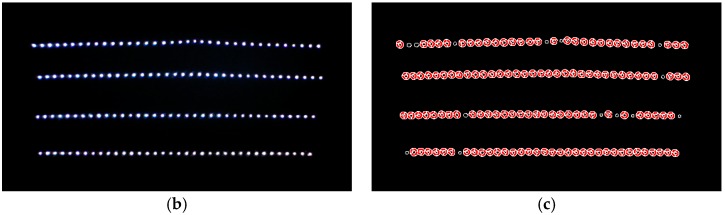
Waxing image analysis following system parameter optimization (waxing force 15 N, dwell time 0.5 s/pt). (**a**) waxing results comparison (proposed system vs. car detailer); (**b**) captured image with the proposed system; (**c**) Hough circle transform results (130 circles).

Figure 17 presents the image analysis results of waxing with the optimal parameters. As can be seen in Figure 17a, the resulting reflection is very close to that in the area waxed by the auto detailer. The sheet metal displayed a mirror-like reflection of the LED light spots, and the number of circles that reached the assessment criterion was 130, which is extremely close to the 132 derived in optimization. Table 8 compares the execution results of the system with those of the auto detailer. Although the optimal parameters were used for waxing, it is still noticeable that the execution time needed by the system was far from that needed by the auto detailer. This is because we used a smaller robotic arm and smaller waxing sponge (diameter 80 mm) for the waxing procedure so as to prevent overburdening the *xyz* platform during operation. In contrast, the sponge used by the auto detailer was 150 mm in diameter, which covered a much larger area, and this resulted in the difference in execution time. The experimental results verify the feasibility of the proposed system and demonstrate that the parameter optimization analysis can further improve actual waxing quality.

## 7. Conclusions

This study developed a robotic waxing system for auto detailing and an image-based assessment method for performance evaluation and improvement. We adopted a depth camera to obtain the color and depth information of the sheet metal and then established waxing paths using image processing and coordinate conversion. In order for the robotic arm to maintain constant waxing force and better compliance, we adopted a position-based impedance control framework to realize waxing force control. With the waxing results of an experienced auto detailer as the standard, we analyzed images of LED point light reflections on the waxed sheet metal to develop a set of assessment criteria for the waxing system. We performed experiments and parameter optimization analysis with two parameters, waxing force and dwell time, which served as the primary impact factors of waxing quality. The results indicate that the proposed system can successfully achieve the waxing quality of an experienced auto detailer and provide reference for further developments on whole automated waxing systems in auto detailing.

## Figures and Tables

**Table 1:** Curve fitting results from the side view of sheet metal (Paths 1 to 6).

# of Path	Path Function (after Cubic Curve Fitting)
Path 1	$z_{1}(y)=-2.86\times 10^{-6}y^{3}+0.0055y^{2}-2.135y-126.0215$
Path 2	$z_{2}(y)=-1.02\times 10^{-5}y^{3}+0.0223y^{2}-15.037y-3154.08$
Path 3	$z_{3}(y)=-5.49\times 10^{-6}y^{3}+0.0117y^{2}-6.9988y-1134.38$
Path 4	$z_{4}(y)=-7.69\times 10^{-6}y^{3}+0.0167y^{2}-10.716y-2051.36$
Path 5	$z_{5}(y)=-7.29\times 10^{-6}y^{3}+0.0156y^{2}-9.7094y-1746.57$
Path 6	$z_{6}(y)=-1.17\times 10^{-5}y^{3}+0.0256y^{2}-17.241y-3607.75$

**Table 2:** Radius distributions of reflected light spots from different camera.

	Samples in Different Range ($\bar{x}=4.40,\sigma =0.20$)		
Camera View (θ)	Total Samples	$\bar{x}\pm \sigma$	$\bar{x}\pm 2\sigma$
15°	144	96 samples	130 samples
30°	144	98 samples	135 samples
45°	144	97 samples	137 samples

**Table 3:** Waxing image assessment criteria adopted in this study.

Estimation of Radius	Criterion Definition
Mean value ($\bar{x}$)	4.4 pixel
Standard deviation (*σ*)	0.2 pixel
Range of circle radius	$\bar{x}\pm 2\sigma$
Average result from Table 2	134 samples
Threshold	10 samples

**Table 4:** Waxing experiment parameter settings.

Waxing Motor Specifications	Waxing Force (From 10 to 19 N)	Dwell Time	Executing Time for all Paths (Approx.)
24 V—850 rpm (30 N-m)	10 N	0.1 s/pt 0.2 s/pt 0.3 s/pt 0.4 s/pt	5~6 min 10~11 min 15~16 min 20~21 min
	13 N		
	15 N		
	17 N		
	19 N		

**Table 5:** Experimental results of different waxing parameters.

Waxing Force	Dwell Time	Executing Time	Estimated Result (Total: 144 Samples)
10 N	0.1 s/pt	5 min 28 s	70 samples
	0.2 s/pt	10 min 35 s	82 samples
	0.3 s/pt	15 min 24 s	89 samples
	0.4 s/pt	20 min 19 s	93 samples
13 N	0.1 s/pt	5 min 36 s	92 samples
	0.2 s/pt	10 min 21 s	102 samples
	0.3 s/pt	15 min 18 s	110 samples
	0.4 s/pt	20 min 30 s	115 samples
15 N	0.1 s/pt	5 min 23 s	110 samples
	0.2 s/pt	10 min 29 s	116 samples
	0.3 s/pt	15 min 34 s	121 samples
	0.4 s/pt	20 min 36 s	127 samples
17 N	0.1 s/pt	5 min 31 s	112 samples
	0.2 s/pt	10 min 33 s	117 samples
	0.3 s/pt	15 min 29 s	115 samples
	0.4 s/pt	20 min 24 s	110 samples
19 N	0.1 s/pt	5 min 36 s	115 samples
	0.2 s/pt	10 min 21 s	117 samples
	0.3 s/pt	15 min 18 s	115 samples
	0.4 s/pt	20 min 30 s	113 samples

**Table 6:** Coefficient table of surface equation.

*p*_00_	−7917	*p*_21_	−76.58	*p*_04_	18140
*p*_10_	3081	*p*_12_	−1923	*p*_50_	0.01935
*p*_01_	−3663	*p*_03_	−8127	*p*_41_	−0.008048
*p*_20_	−471.6	*p*_40_	−1.324	*p*_32_	−4.448
*p*_11_	915.7	*p*_31_	2.224	*p*_23_	12.24
*p*_02_	9295	*p*_22_	159.7	*p*_14_	−255.9
*p*_30_	35.63	*p*_13_	−83.42	*p*_05_	−11600

**Table 7:** Constraints of optimization.

Optimization Parameters of the Waxing System	Lower and Upper Bounds
Force (N)	From 10 N to 17 N
Dwell time (s/pt)	From 0.1 s/pt to 0.5 s/pt

**Table 8:** Comparison of waxing results (proposed system: waxing force 15 N, dwell time 0.5 s/pt).

	Executing Time	Estimated Result (Total: 144 Samples)
Car detailer	About 15 min	134 samples
The waxing system	25 min 16 s	130 samples
